# Detection of underdiagnosed concurrent branch retinal artery occlusion in a patient with central retinal vein occlusion using spectral domain optical coherence tomography

**DOI:** 10.1186/1471-2415-14-91

**Published:** 2014-07-12

**Authors:** Anushavan Karapetyan, Pingbo Ouyang, Luo Sheng Tang, Jiexi Zeng, Michele Dominique Li Ying

**Affiliations:** 1 Department of Ophthalmology, The Second Xiangya Hospital, Central South University, 139 Renmin Middle Road, Changsha 410011, China

**Keywords:** Branch retinal artery occlusion, Central retinal vein occlusion, Spectral domain optical coherence tomography

## Abstract

**Background:**

Combined branch retinal artery and central retinal vein occlusion is a rare condition that has been infrequently reported. This case report, aside from reporting the above-mentioned condition, highlights the importance of performing spectral domain optical coherence tomography in establishing a complete diagnosis, especially in uncertain and complicated cases. We also present spectral domain optical coherence tomography findings of a case of combined unilateral simultaneous central retinal vein and branch retinal artery occlusion.

**Case presentation:**

We present a single case of an initially missed, unilateral branch retinal artery occlusion combined with central retinal vein occlusion in a 51-year-old female Chinese patient without a significant past medical history, who experienced sudden, painless vision diminution in her right eye eleven days prior to presentation. She eventually recovered visual acuity to 0.60, despite having presented with poor vision.

**Conclusion:**

Combined unilateral central retinal vein and branch retinal artery occlusion may occur in patients with no medical history of arterial hypertension and diabetes mellitus and can achieve a relatively good visual outcome. This case reaffirms the significance of performing a spectral domain optical coherence tomography examination in patients suffering from central retinal vein occlusion with suspicion of unilateral simultaneous branch retinal artery occlusion to identify the affected pathological areas.

## Background

Central retinal vein occlusion (CRVO) and branch retinal artery occlusion (BRAO) are two different types of retinal vascular occlusions. CRVO is a common retinal vascular disorder that arises from a blockage of the central retinal vein. As a consequence of the blockage, stagnant blood gradually leaks out through the vein walls and leads to blurred vision. BRAO refers to an obstruction or blockage of one or multiple branch retinal arteries resulting in a severe loss of vision, the area and degree of which is associated with the distribution of the occluded branch retinal artery. Histopathologically, acute BRAO results in ischemia in the corresponding retinal quadrant marked by inner retinal edema in the initial stages and atrophy in long-standing cases [1]. The combination of the two aforementioned diseases is rare, despite the fact that these two types of ocular vascular obstructions share many common underlying systemic conditions, such as cardiovascular atherosclerotic disease, arterial hypertension, diabetes mellitus, toxoplasmosis, sarcoidosis, Behçet's disease [2], coagulopathies [3]*,* systemic lupus erythematosus and anti-phospholipid syndrome [4], and homocysteinemia [5], which result in severe loss of vision and impairment of the patient’s quality of life.

In this report, we describe a single case of combined unilateral CRVO and BRAO and emphasize the efficacy of spectral domain optical coherence tomography (SD-OCT) examination in this type of case.

## Case presentation

**Figure 1:**
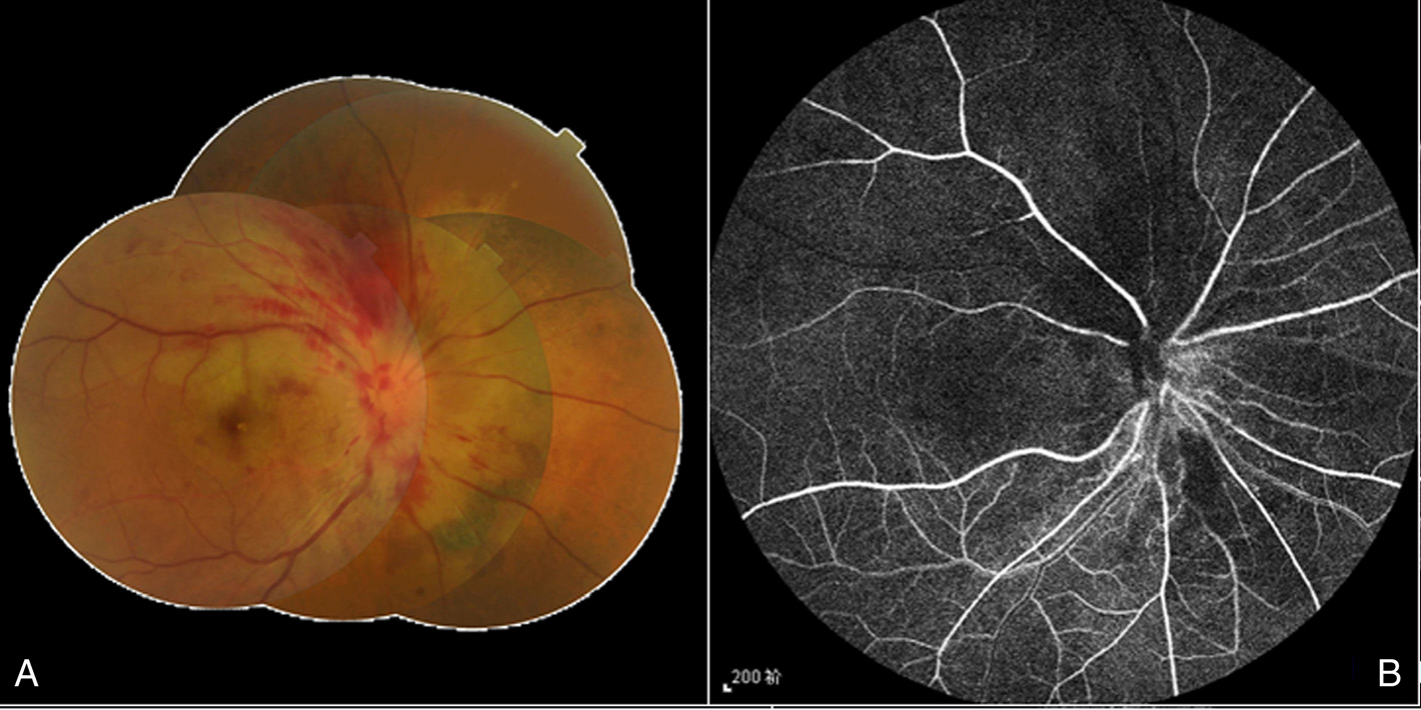
**Color fundus photo taken at presentation. (A)**. Clearly demarcated pale retina in the distribution of the superior temporal artery, swollen optic disc, tortuous and dilated veins. Owing to late presentation and traditional Chinese medicine given initially, the typical blot/dot hemorrhages are almost resolved and flame-shaped effusion of blood can be observed. FA image of the arterial phase 16 seconds after fluorescein dye injection **(B)**. Delayed filling of the superotemporal artery and hypofluorescence in the surrounding artery area can be seen.

**Figure 2:**
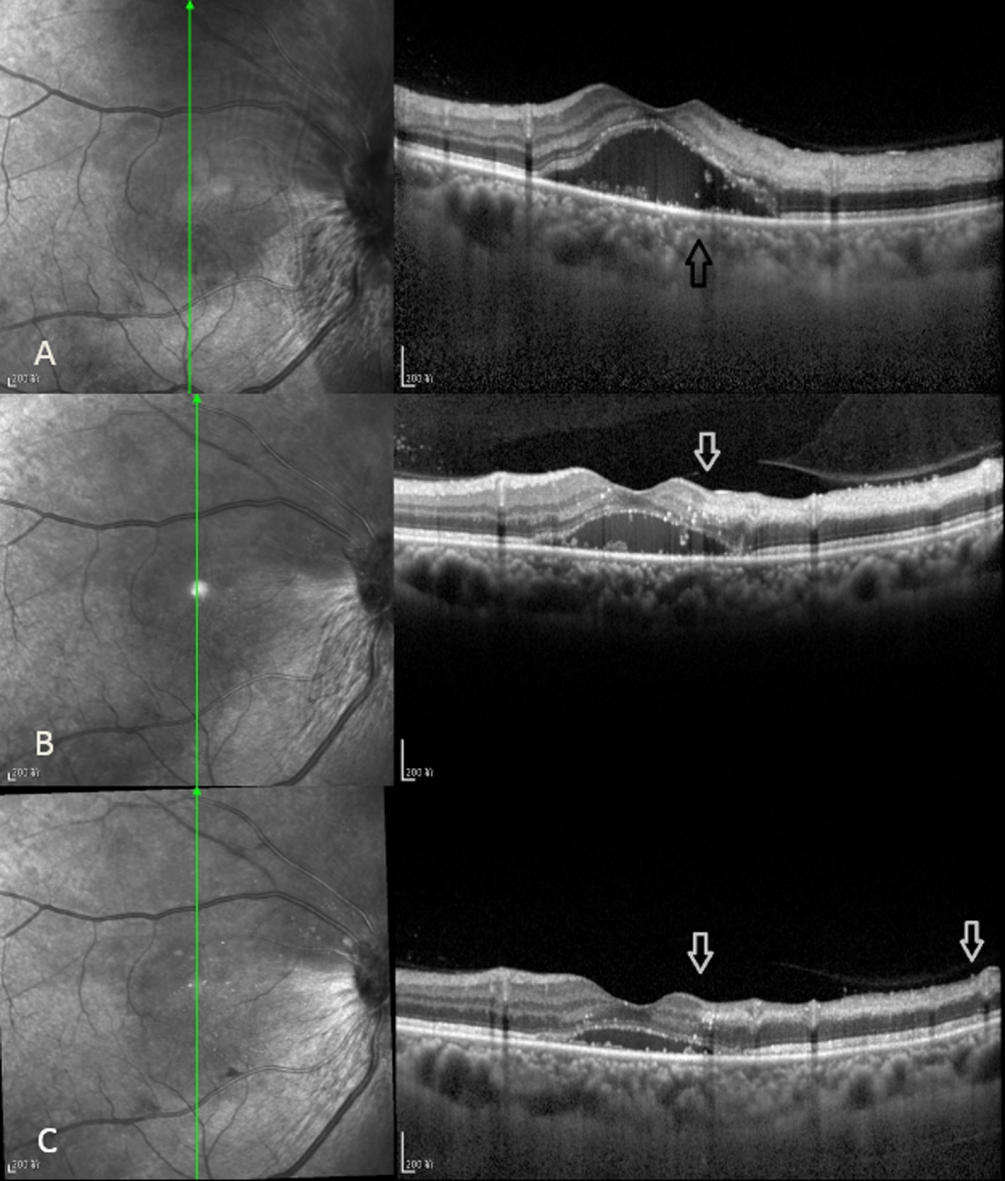
**SD-OCT image taken at presentation. (A)**. Macular edema and macular detachment, boundary between hyper and normo-reflective retinal regions (arrow). SD-OCT image taken 1 week later at first follow-up visit **(B)**. Decreased volume of submacular fluid and macular detachment. Well-defined demarcation line between affected and unaffected inner retinal layers (arrow). SD-OCT image taken 2 weeks later at second follow-up visit **(C)**. Further decreased submacular fluid, macular detachment, atrophied inner layers of the superior retina (between arrows).

A 51-year-old female Chinese patient without arterial hypertension, diabetes mellitus or significant past ophthalmic history presented to our hospital complaining of painless, suddenly impaired vision in her right eye for 11 days. Prior to that, she had been hospitalized at the local county hospital with visual acuity of 0.06 and 0.80 in the right and left eyes, respectively. Based on the results of ophthalmologic, fundus and fluorescein angiography (FA) examinations, clinical diagnosis of CRVO was established and traditional Chinese medicine was prescribed to activate blood circulation and decrease blood stasis. Upon presentation to our hospital, the anterior segment examination under a slit lamp biomicroscope (SLE) was unremarkable in both eyes, and visual acuity of 0.10 and 0.80 was revealed in the right and left eyes, respectively. Intraocular pressure was in the normal range. A dilated fundus evaluation demonstrated an edematous macula, tortuous and dilated retinal veins with radially patterned hemorrhages, blurred and elevated disc margins in her right eye, and retinal paleness in the upper region of the macula (Figure 1A). On the basis of these signs, combined with the results from FA performed at the local hospital, the diagnosis of CRVO was reconfirmed. The FA examination was repeated at our hospital, which showed signs suggestive of BRAO (Figure 1B) and the patient was recommended to undergo SD-OCT examination. SD-OCT showed macular edema with a shallowly detached fovea, an edematous retina in its all sections, subfoveal liquid, a detached peripapillary retina, intact inner segment-outer segment (IS-OS) line and a strong reflected signal from thickened inner layers of the superior retina (ILSR) in contrast to the inferior retina, suggesting the possibility of merging BRAO in this region (Figure 2A). A retrospective view of the fundus photographs showed that, in addition to radial hemorrhages in the fundus of the right eye, a clearly demarcated pale area at the superior region of the retina existed. Because of the obvious CRVO manifestation, this region had been neglected during the previous examinations. Subsequently, the complete clinical diagnosis of combined unilateral CRVO and BRAO was made. The patient had neither medical history nor signs and symptoms of cardiovascular, hematological, systemic and parasitic diseases. The instrumental examinations, such as chest X-ray and echocardiography, did not reveal any pathology and blood and urine laboratory test results, namely enzyme-linked immunosorbent assay (ELISA), polymerase chain reaction (PCR), plasma and urine homocysteine quantitative tests, full blood count were within the normal range. The ELISA and PCR testes were used to screen for β2-glycoprotein 1 dependent anticardiolipin (ACA), immunoglobulin G antibodies and Toxoplasma gondii B1 gene. Owing to a late presentation (>24 hours), the only treatment offered was one periocular injection of triamcinolone acetonide (40 mg). One week later at the first follow-up visit, the patient had visual acuity of 0.40 and 0.80 in the right and left eyes, respectively. A SD-OCT examination showed decreased volume of the accumulated fluid in the sub-neuroepithelial space as well as weakened reflected signal from the ILSR and reduced retinal thickness (Figure 2B). Two weeks later at the second and last follow-up visit, SD-OCT examination showed a further decrease of the sub-neuroepithelial fluid volume, reduced nonuniform reflected signal from and atrophy of the ILSR (Figure 2C). The patient's visual acuity was 0.60 and 0.80 in the right and left eyes, respectively.

**Figure 3:**
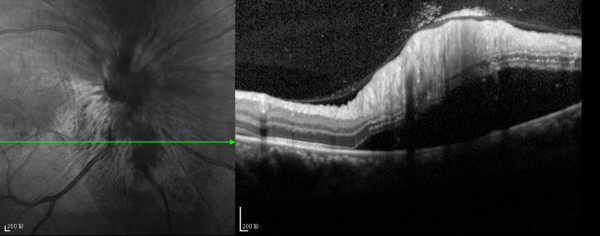
**SD-OCT image taken at presentation.** Detached peripappillary retina below the optic disk.

The SD-OCT features of combined unilateral CRVO and BRAO in our case were:

1. hyperreflectivity and increased thickness of the nerve fiber and inner retinal layers in the superior ischemic retina compared to the inferior unaffected area near the BRAO retina;

2. decreased reflectivity of the outer retinal layers in the superior half, probably due to an optical shadowing effect;

3. macular edema and serous macular detachment typical for CRVO;

4. no cystoid macular edema was observed either on presentation or at follow-up visits;

5. a well-defined demarcation line between the affected and unaffected areas near the BRAO retina6. peripapillary retinal detachment (Figure 3)

## Discussion

Combined unilateral simultaneous CRVO and BRAO is a rare condition, which if incomplete, has been proposed to be deemed as one entity. In patients with a combined concurrent CRVO and BRAO, an embolic compound has not been found, suggestive of the secondary nature of BRAO due to compression of the artery because of the obviously swollen optic nerve and/or central retinal vein resulting from CRVO [6]. This is the main distinction from an isolated BRAO, which often is caused by embolic events. The patients with combined BRAO and incomplete CRVO have better visual outcomes than do those suffering from other types of combination retinal artery and vein occlusions, and almost 80% of the patients recover final visual acuity greater than 0.50, as occurred in our case [1,2].

In our case, we performed the assisting diagnostic procedure with SD-OCT in addition to FA, in order to establish the final diagnosis of combined unilateral CRVO and BRAO. This approach is optimal for patients with a questionable diagnosis of combined unilateral CRVO and BRAO. SD-OCT has quite recently been introduced to the medical world and has revolutionized the ocular imaging service. It is a cutting-edge technique using a significantly faster, non-mechanical technology providing non-invasive acquisition of detailed; i.e., up to 5 microns resolution, *in-vivo* histological changes in the retina [1,7].

These anatomical changes are the result of the denaturation of intracellular proteins, with the accumulation of intracellular fluid and consequent cellular necrosis. We propose that the name ‘retinal edema’ should not be used for this clinical condition, as the extravascular fluid is found in the intracellular space, and we suggest that the term ‘acute ischemic retinopathy’ is more appropriate [8].

Preservation of the integrity of the IS-OS line and the external limiting membrane has been reported to have prognostic value in cystoid macular edema due to vascular occlusions [9]. Presence of an intact IS-OS line at the fovea in our patient suggested the preservation of structural integrity of the photoreceptor layer at the fovea, contributing to the recovery of relatively good visual acuity.

Throughout the follow-up visits, atrophy of the superior retina was observed.

## Conclusion

Combined unilateral CRVO and BRAO may occur in patients without a significant past medical history. SD-OCT is an efficient method for establishing a combined unilateral CRVO and BRAO diagnosis. Timely use of SD-OCT facilitates fast and precise diagnosis of the aforementioned condition, ensuring prompt treatment. In our case, although the patient presented with poor visual acuity, we can report the presence of an intact IS-OS line on SD-OCT, thanks to which a good visual outcome was attained.

### Consent

Written informed consent was obtained from the patient for publication of this case report and any accompanying images. A copy of the written consent is available for review by the Editor of this journal.

## Abbreviations

BRAO: Branch retinal artery occlusion; CRVO: Central retinal vein occlusion; SD-OCT: Spectral domain optical coherence tomography; ILSR: Inner layers of superior retina; IS-OS: Inner segment-outer segment; FA: Fluorescein angiography; ELISA: Enzyme-linked immunosorbent assay; PCR: Polymerase chain reaction.

## Competing interests

The authors declare that they have no competing interest.

## Authors’ contributions

TLS critically revised the manuscript and gave final approval of the version to be published, AK designed, drafted the manuscript in English and made the submission, PO drafted the manuscript in Chinese and examined the patient, JZ and LYMD helped to draft and translate the Chinese manuscript, case history and examination reports and were involved in the clinical and scientific discussion of the case. All five authors revised the manuscript and made intellectual contributions. All authors read and approved the final manuscript.

## Pre-publication history

The pre-publication history for this paper can be accessed here:

http://www.biomedcentral.com/1471-2415/14/91/prepub
